# P-1515. Outcomes of Patients with Piperacillin/Tazobactam-Non-Susceptible and Ceftriaxone-Susceptible Enterobacterales Bacteremia

**DOI:** 10.1093/ofid/ofae631.1684

**Published:** 2025-01-29

**Authors:** Michelle Lee, Emily O’Neill, Cheston Cunha, Sara Geffert, Chris Nering, Emerald O’Rourke

**Affiliations:** Rhode Island Hospital, Providence, Rhode Island; Lifespan, Providence, Rhode Island; Warren Alpert Medical School of Brown University, Providence, Rhode Island; Lifespan / The Warren Alpert Medical School of Brown University, Providence, Rhode Island; Lifespan, Providence, Rhode Island; Lifespan, Providence, Rhode Island

## Abstract

**Background:**

Piperacillin/tazobactam (TZP)-non-susceptible (NS) but third-generation cephalosporin (3GC)-susceptible (S) *Escherichia coli* and *Klebsiella pneumoniae* are increasingly reported in the clinical setting. This phenotype is associated with hyperproduction of β-lactamases, including TEM-1 and SHV-1. Tailoring therapy to a 3GC in these cases may decrease carbapenem use and promote antimicrobial stewardship. However, the optimal definitive β-lactam regimen for this phenotype is unknown. This retrospective case series describes the clinical outcomes and antibiotic prescribing in patients with TZP-NS and ceftriaxone (CRO)-S Enterobacterales bacteremia at a three hospital health system.

**Figure:**
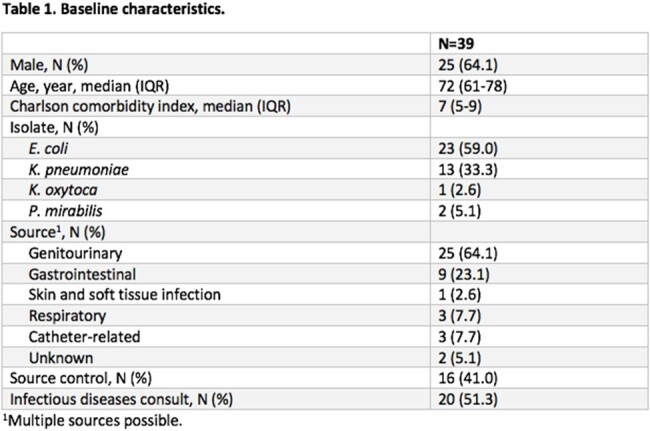

**Methods:**

We identified patients >18 years of age hospitalized with TZP-NS and CRO-S *E. coli*, *K. pneumoniae*, *Klebsiella oxytoca*, and *Proteus mirabilis* bacteremia between February 2020 and March 2024. TZP-NS and CRO-S were defined per the 2024 Clinical and Laboratory Standards Institute (CLSI) susceptibility breakpoints: TZP-S if minimum inhibitory concentration (MIC) ≤ 8/4 mg/L, TZP-NS if MIC ≥ 16/4 mg/L; CRO-S if MIC ≤ 1 mg/L, CRO-NS if MIC ≥ 2 mg/L. We included only the first isolate per patient over the study period. Antibiotic data were collected for each patient during hospitalization. We defined empiric antibiotics as the agents with the longest duration of therapy (DOT) prior to susceptibilities. Composite clinical outcomes were 30-day all-cause mortality, or 30-day readmission/emergency department (ED) visit.

**Figure:**
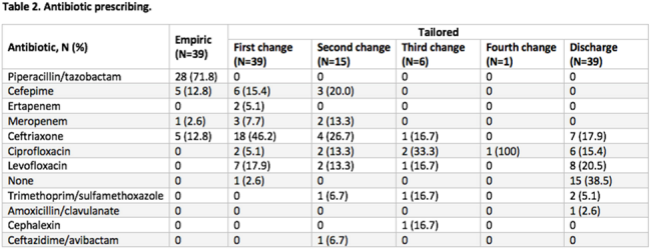

**Results:**

Thirty-nine patients were included in the study (Table 1). The median number of different antibiotics patients received was 3 (interquartile range [IQR] 2-3), and the median total antibiotic DOT was 14 days (IQR 9.5-17.5 days) (Table 2). Thirteen patients experienced a clinical outcome (33.3%) (Table 3). Of the patients who experienced a clinical outcome, 46.2% (6/13) received ceftriaxone during tailored therapy (median 4.5 days, IQR 3.3-7.3 days).

**Figure:**
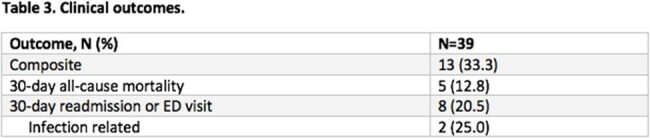

**Conclusion:**

In this case series, most patients received TZP empirically but were tailored to CRO upon culture susceptibility availability. Larger studies are necessary to elucidate if ceftriaxone is appropriate for definitive treatment of TZP-NS and CRO-S *E. coli*, *K. pneumoniae*, *K. oxytoca*, and *P. mirabilis* bacteremia.

**Disclosures:**

**All Authors**: No reported disclosures

